# Surgical Management of Massive Irreparable Cuff Tears: Latissimus Dorsi Transfer for Posterosuperior Tears

**DOI:** 10.1007/s12178-020-09659-3

**Published:** 2020-07-13

**Authors:** Karl Wieser, Lukas Ernstbrunner, Matthias A. Zumstein

**Affiliations:** 1 Department of Orthopedics, Balgrist University Hospital, University of Zurich, Forchstrasse 340, CH-8008 Zurich, Switzerland; 2 Shoulder, Elbow & Orthopaedic Sports Medicine, Orthopaedics Sonnenhof; Inselspital, University of Berne, Bern, Switzerland; 3 SportsClinicNumber1, Bern, Switzerland

**Keywords:** Latissimus dorsi transfer, Tendon transfer, Arthroscopically assisted, Massive rotator cuff tear, Posterosuperior rotator cuff

## Abstract

**Purpose of Review:**

This review aims to describe the role of the latissimus dorsi transfer (LDT) for patients with irreparable posterosuperior rotator cuff tears (RCTs).

**Recent Findings:**

Historically, the LDT has been performed as an open (double-incision) procedure for neurologically intact, relatively young patients with irreparable posterosuperior RCTs with disabling loss of active external rotation with or without impaired active elevation. The transferred tendon reconstitutes the posterior rotator cuff and force couple, respectively and thus has the potential to function effectively as an external rotator and humeral head depressor. Long-term results of the open technique have demonstrated in the majority of patients substantial and durable improvements in shoulder function and pain relief at the 10-year benchmark. With the advancements of arthroscopic surgery, the LDT was expanded to an arthroscopically assisted procedure with promising short-term results. In addition to adequate technical performance, the success of the procedure depends on preoperative factors, such as exclusion of glenohumeral osteoarthritis and acromial acetabularization; intact or reparable subscapularis tendon; intact (or hypertrophic) teres minor muscle; adequate preoperative activity of the latissimus dorsi; and normal or mild impairment of overhead function.

**Summary:**

The LDT (open or arthroscopically assisted) is a reliable treatment option for patients with massive, irreparable posterosuperior RCTs with disabling loss of active external rotation, with or without diminished overhead function and without advanced glenohumeral osteoarthritis. Precise patient selection is of tremendous importance in the success of the LDT.

## Introduction

The generated forces of co-contracting rotator cuff muscles stabilize the humeral head within the glenoid concavity providing a stable fulcrum for optimal force transmission of the deltoid, rotator cuff, and periscapular muscles. Rupture of the rotator cuff tendons is common and might cause disruption of this delicately balanced force couple of anterior (subscapularis) and posterior (infraspinatus and teres minor) rotator cuff. With tear progression, joint compression forces and stability within the glenohumeral joint decrease, leading to diminished range of motion and strength.

The incidence of asymptomatic tears in patients over 60 years of age is high [1, 2], and up to 40% of all rotator cuff tears (RCTs) are classified as massive [3–5]. A general definition of massive RCTs (mRCTs) includes involvement of two or more tendons [6]. If the RCT involves the posterosuperior cuff, typical functional deficits are weakness of abduction and external rotation with diminished overhead function [7, 8••]. However, the clinical presentation of patients with posterosuperior mRCTs can vary widely from slight weakness and pain to pseudoparesis or even paralysis (PP) [9–11] with loss of control of the arm in space [12, 13]. The hallmarks of chronic RCTs are atrophy [14], fatty infiltration [15], and myotendinous retraction [16], which can be assessed and quantified on MRI- or CT arthrography. Conventional radiographic changes associated with massive posterosuperior RCTs are cranial migration of the humeral head and progressive glenohumeral osteoarthritis (OA) [12, 13].

If fatty infiltration of the torn muscles is greater than stage 2 according to the Goutallier classification [15], and the acromiohumeral distance is less than 7 mm, the probability for successful structural rotator cuff repair becomes so low that these mRCTs are generally considered irreparable (functionally irreparable rotator cuff tear (FIRCT)) [17]. With loss of the compressive force yielded through the rotator cuff, eccentric loads are placed upon the glenoid leading to erosion and early OA. Treatment should aim to restore more centred joint mechanics, which theoretically could slow the progression of OA, and to reconstitute the posterior rotator cuff and force couple, and thus shoulder function; these goals are particularly important in younger patients with high demands. The latissimus dorsi transfer (LDT) has been described to function as such external rotator and humeral head depressor [18] and is a reliable treatment option to alleviate pain and functional disability associated with irreparable posterosuperior RCTs in younger patients [7, 8••, 19–21].

## Clinical Presentation

Clinical findings in patients with FIRTC can vary widely from slight weakness and pain to substantial loss of active shoulder function. Large tears after trauma oftentimes present with sudden loss of shoulder function [22], whereas chronic RCTs are usually associated with slow and progressive loss of function with increasing pain. Not all RCTs are symptomatic; if the remaining cuff and periscapular muscles are strong enough to compensate the torn rotator cuff tendons, FIRCTs can be associated with almost normal shoulder function and no pain.

The main disability experienced by many patients with a FIRCT is weakness with the arm away from the body. Posterosuperior FIRCTs typically cause weakness of elevation and external rotation [23]. There is a wide range of such *weakness* which ranges from slightly, nonquantifiable weakness to complete loss of active function of the respective muscle, so-called pseudoparalysis. The definition of pseudoparalysis is still under debate. Some surgeons use the term pseudoparalysis when there is no glenohumeral motion, and very limited elevation is provided through scapulothoracic motion only; pseudoparesis could be defined by the pain-free inability of actively abduct to 90° with normal passive range of motion and absence of neurologic impairment [17]. Conversely, pseudoparalysis of external rotation is defined as complete loss of active external rotation strength in the presence of unrestricted passive external rotation and absence of neurologic impairment, which can be assessed using the Neer drop-arm sign [24], the external rotation lag sign [25], or the hornblower’s sign [8, 26]. A functioning teres minor muscle is one of the factors associated with better outcome after LDT, and its function can be assessed through the so-called external rotation position 2—the arm in 90° of abduction—while evaluating external rotation against resistance. A positive hornblower’s sign is therefore likely associated with tear extension from the infraspinatus into the teres minor, which might negatively influence the final outcome of a LD tendon transfer.

Examination of the shoulder should also include subscapularis function (i.e. lift-off, belly-press, and bear hug tests). Obvious dynamic anterosuperior subluxation of the humerus upon resisted abduction is suggestive of an irreparable posterosuperior with additional subscapularis tear. In case of complete pseudoparalysis and/or a dynamic anterosuperior escape of the humeral head, which is most likely associated with an irreparable subscapularis tear [27–29], a LDT will not be able to substantially improve shoulder function and is therefore not indicated in such severely functionally impaired shoulders.

## Imaging

True anteroposterior radiographs are useful to assess glenohumeral OA [30] and static superior migration of the humeral head. A static, not reducible, chronic acromiohumeral distance of < 7 mm on plain anteroposterior radiographs with the arm in neutral rotation has been reported to be strongly associated with FIRCTs [10, 17]. The critical shoulder angle (CSA) should be assessed as well, as a wide lateral extension of the acromion in the coronal plane is associated with worse outcome after LDT [20••]. Axillary lateral and scapular lateral radiographs should be assessed for anterior or posterior subluxation of the humeral head.

**Fig. 1:**
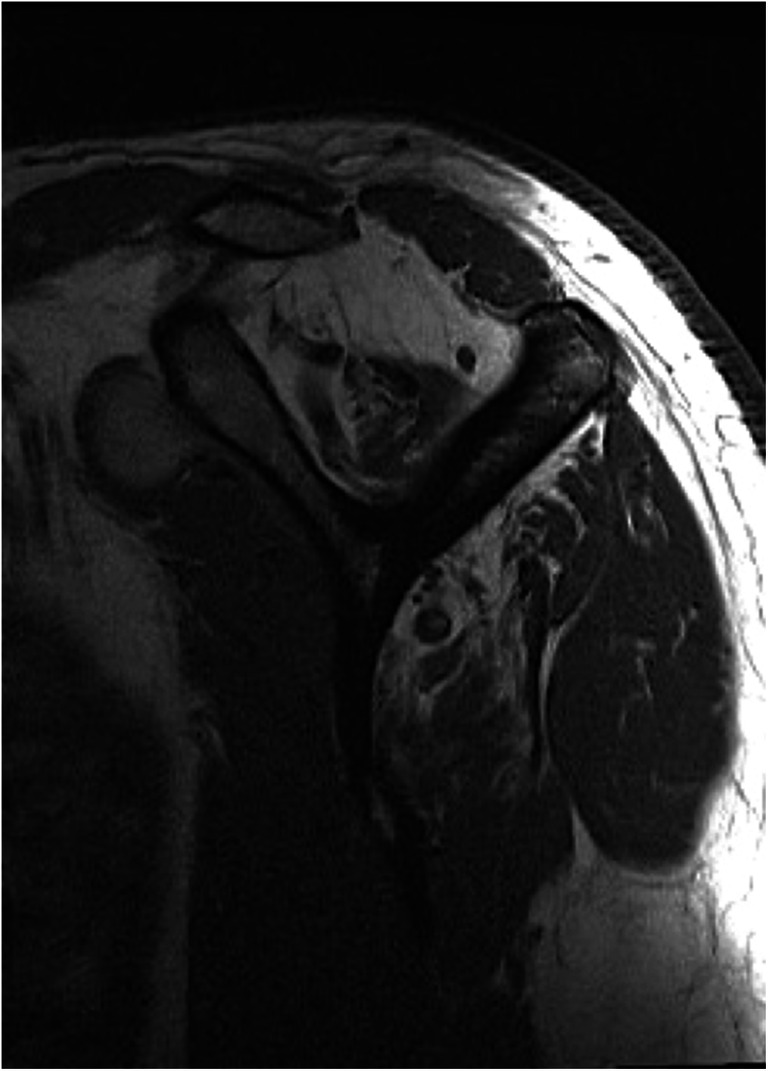
Parasagittal T1-weighted MR imaging showing advanced fatty infiltration of the supraspinatus (with atrophy) and infraspinatus muscles. Muscles of the subscapularis and teres minor show no degeneration

MR imaging is useful to assess the quality of rotator cuff tendons and its muscles. Fatty infiltration of the supraspinatus and infraspinatus muscles greater than stage 2 according to Goutallier [15] or the MRI modification thereof [31] is known to be associated with irreparability of RCTs [32, 33] (Fig. 1).

Integrity of the subscapularis and teres minor tendons and quality of the corresponding muscles should be assessed as they either need to be addressed intraoperatively and/or influence the clinical outcome.

## Treatment Options

### Nonoperative Treatment

Although there is no evidence that conservative treatment alters the natural history of FIRCTs, good clinical results after conservative treatment have been reported [34]. If the cuff tear is irreparable, as long as functional impairment and pain are tolerated, nonoperative management including physiotherapy, nonsteroidal anti-inflammatory medications and corticosteroid injections can be considered.

### Surgical Treatment

Patients with FIRCT who fail nonoperative management can be considered for surgical treatment, which may include arthroscopic debridement with biceps tenotomy or tenodesis, partial RCT repair with or without augmentation, superior capsular reconstruction, tendon transfers, and reverse total shoulder arthroplasty (RTSA) [8, 35–38]. Arthroscopic debridement and partial repair have been proved to provide reliable pain relief [39, 40], but these procedures do not seem to halt or substantially decrease the progression of glenohumeral OA [40, 41]. There is good evidence to suggest that successful repair of massive RCTs provides excellent clinical long-term results and that progression of glenohumeral OA can be decelerated [6, 42–44]. RTSA has been shown to provide satisfactory long-term results for massive, irreparable RCTs in elderly and even in younger patients after failed rotator cuff repair [10, 11, 17, 45]. However, the biomechanics of RTSA does not allow for restoration of a good arc of external rotation in patients with pseudoparalysis of external rotation. Furthermore, in young and active patients without signs of OA, joint-preserving options such as tendon transfers should be considered beforehand.

When considering a tendon transfer, the following principles should be fulfilled: (1) the transferred muscle should be expendable without compromising the donor site, (2) the transferred and recipient muscle should have a similar excursion and tension, and (3) the line of pull (vector) of the transferred tendon and recipient muscle should be as similar as possible [18].

### Latissimus Dorsi Transfer

#### Indications

The success of the LDT depends on proper patient selection. The subscapularis tendon should be intact or reparable [20••]. The inferior functional results after LDT with a dysfunctional subscapularis are explained by insufficient centring of the humeral head during abduction and elevation [46•]. Inferior results have also been observed when chronic pseudoparalysis of active anterior elevation was present, and also when the teres minor showed advanced fatty infiltration [19, 47]. Failure of the LDT can be anticipated when glenohumeral OA or acromial acetabularization are present [48•]. Details about relative and absolute contraindications for LDT are summarized in Table 1.

**Table 1:** Relative and absolute contraindications for latissimus dorsi tendon transfer

Relative contraindications	Absolute contraindications
Advanced age	Deltoid insufficiency
Severe fatty infiltration of TMin	Advanced glenohumeral osteoarthritis
SSC repair under tension	Irreparable SSC tear
Poor bone quality	Infection
Poor compliance	Complete pseudoparalysis of abduction
	Dynamic anterosuperior escape

*TMin*, teres minor; *SSC*, subscapularis

#### Anatomy and Biomechanics

The latissimus dorsi is triangle-shaped and has a broad origin from the sacrum, posterior iliac crest, spinous processes of T7-L5, and ribs 9–12. It inserts onto the intertubercular groove between the pectoralis major anteriorly and the teres major posteriorly [49•]. It is innervated by the thoracodorsal nerve (C6-C8), from the posterior cord of the brachial plexus. The thoracodorsal artery provides its blood supply. The thoracodorsal artery and nerve run together as a neurovascular pedicle, which enters the muscle approximately 2 cm medial to the musculotendinous border [50•]. Proximal to the insertion of the latissimus dorsi runs the axillary nerve. The radial nerve runs inferior and medial to the latissimus dorsi insertion.

The latissimus dorsi internally rotates, adducts, and extends the humerus. When the latissimus dorsi tendon is transferred to the greater tuberosity for the management of a FIRCT, it reconstitutes the posterior rotator cuff and force couple, respectively. Thus, it has the potential to function effectively as an external rotator and humeral head depressor that is counterbalanced by the deltoid and intact subscapularis [7, 8, 18].

#### Surgical Technique

In the original description of Gerber et al. [8••], an open double-incision approach involved an approximately 12 cm long superior approach to the rotator cuff with the sagittal incision spanning from the scapular spine to 5 cm ventral to the anterior acromion. The incision is just medial and parallel to the lateral acromion, and the middle deltoid is detached with a small bone chip from the lateral acromion. The remaining rotator cuff is evaluated and repaired when feasible.

With the arm in full elevation, a second L-shaped incision of approximately 20 cm is performed just over the anterior border of latissimus dorsi muscle belly in the axilla. The latissimus dorsi muscle lies anterior to the teres major, and the interval between these two muscles is dissected. The long and broad but very thin tendon of the latissimus dorsi is then sharply released from the humerus and reinforced with two high-strength sutures. A passage between the posterior deltoid and the teres minor is established for the tendon transfer. The latissimus tendon is transferred through the passage and secured at the anterior portion of the greater tuberosity with the arm in 45° of abduction and 45° of external rotation. The middle deltoid is repaired to the lateral acromion using transosseous techniques.

A less invasive technique is the posterior single-incision approach described by Habermeyer et al. [51•]. The main advantage of this procure is that the deltoid muscle is not detached, and the posterior rotator cuff is directly visualized through a posterior V-shaped incision. The main disadvantage is that the anterosuperior rotator cuff cannot be visualized or repaired when necessary.

Recently, with the advancements of arthroscopic surgery, the LDT was expanded to an arthroscopically assisted [52•] or even all-arthroscopic procedure [53•].

With the arthroscope in the posterior standard portal, the anterior portion of the greater tuberosity is prepared for LDT fixation. Then with the arthroscope in the lateral portal, the passage between the deltoid and the teres minor is established with the shaver and radio frequency ablation devices.

**Fig. 2:**
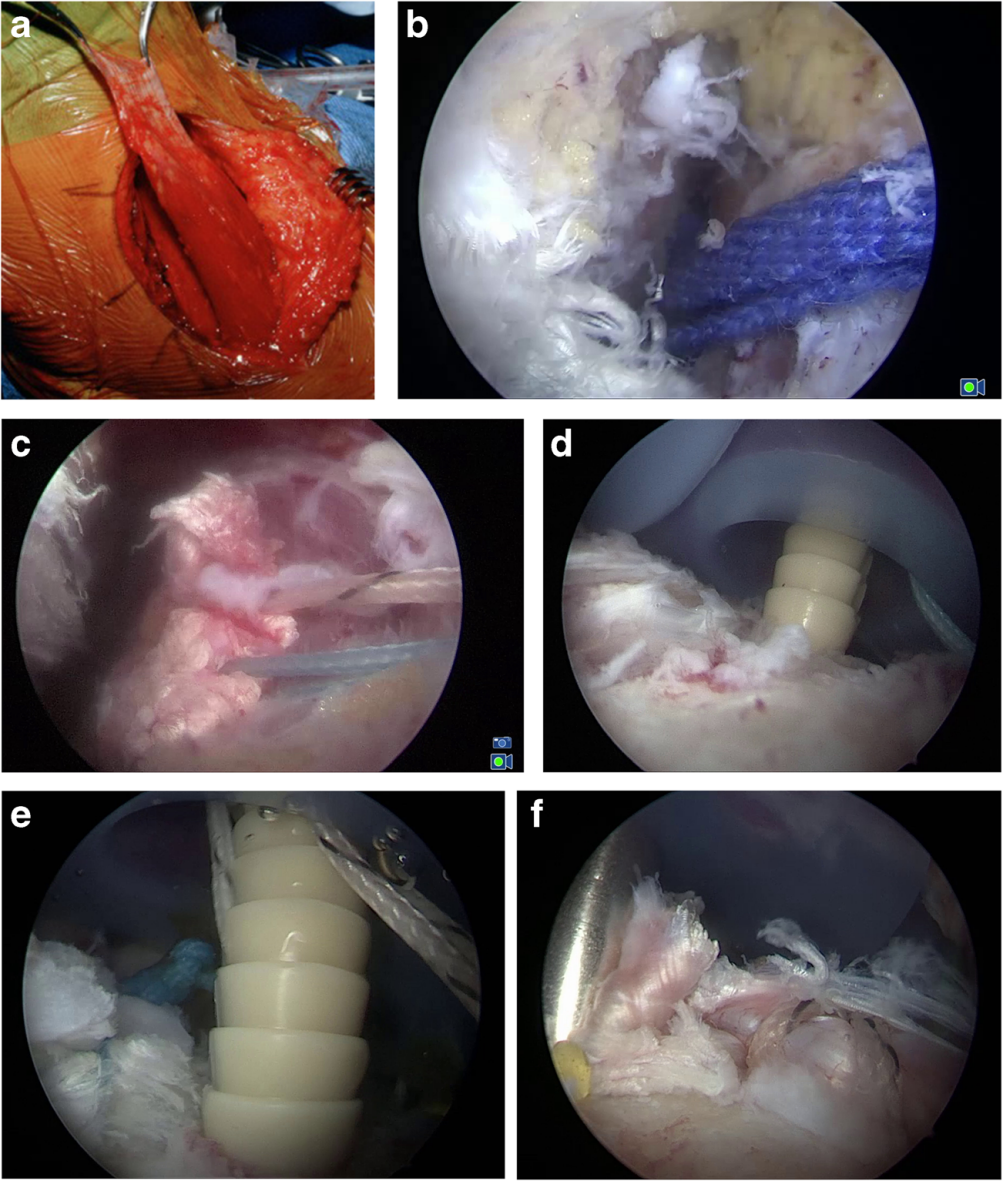
**a** Inraoperative photo documentation of latissimus dorsi tendon transfer (LDT). Through an open axillary approach, the long and broad but very thin tendon of the latissimus dorsi is sharply released from the humerus and reinforced with two high-strength sutures. **b** A passage between the posterior deltoid and the teres minor is established arthroscopically (with the scope in the lateral portal), and a shuttling band (blue) is positioned in the interval for passage of the LDT. **c** The reinforced latissimus tendon is then shuttled through the passage to the prepared anterior portion of the greater tuberosity. **d** and **e** Through two cannulas in each of the two high anterolateral portals, the tendon is refixated with at least two knotless PEEK anchors to the anterior portion of the greater tuberosity. **f** The tendon is tightened through the knotless anchors until it reaches the anchor holes

A shuttling suture or tape is positioned in the interval for later passage of the harvested latissimus dorsi tendon. Finally, after axially harvesting and passing, the tendon is fixed with at least two knotless anchors to the anterior portion of the greater tuberosity (Fig. 2).

One disadvantage of the all-arthroscopic procedure is the inability to perform a proper release of the muscle belly from its connective tissue, making it almost impossible to transfer the tendon to the desired fixation point at the anterior part of the greater tuberosity and leading to a high tension of the tendon-to-bone fixation. In addition, the all-arthroscopic LDT is associated with a very steep learning curve.

The authors therefore prefer the arthroscopic-assisted LDT as it offers some potential benefits over the open technique: (1) iatrogenic deltoid insufficiency can be avoided and the remaining posterosuperior rotator cuff as well as subscapularis tears can be easily diagnosed and repaired. (2) The passage of the tendon between the posterior deltoid muscle and the teres minor can be prepared and visualized with the arthroscope, making it easier to define this interval in comparison with the axillary incision. (3) If desired, the release of the latissimus dorsi tendon can be performed arthroscopically, therefore reducing the axillary incision length, which is then only needed for release of the muscle belly and identification of the intermuscular passage.

Postoperatively, the shoulder is positioned on an abduction brace for 6 weeks. Postoperative physiotherapy is started immediately, with only passive range of motion for the first 6 weeks. After 6 weeks, the abduction brace is discontinued and active range of motion is initiated. After 3 months, gradual strengthening exercises can be initiated with unrestricted strengthening exercises added after 6 months.

#### Clinical and Radiographic Outcome

Long-term results of the open technique of Gerber et al. demonstrated durable improvements in shoulder function and pain relief, with 74% of the patients rating their subjective outcome as good to excellent at the 10-year benchmark [20••]. The postoperative subjective shoulder value averaged at 70% and mean active external rotation was improved from 18° to 30°. Also, active overhead function was significantly improved 10 years postoperatively and averaged beyond 120°. In this series, 32 patients had an increase of the SSV of more than 30%, whereas 14 patients had an increase of the SSV of less than 30% and were considered to have an unsatisfactory outcome. Those patients with a poor outcome had more fatty degeneration of the teres minor, a higher rate of subscapularis insufficiency, and a higher rate of an inactive LDT. Further subgroup analysis showed that patients with a higher CSA had significantly lower subjective and objective outcome scores. Patients with a CSA of less than 36° scored with 91% relative Constant score, significantly higher than the subgroup with a CSA of more than 36°, which had 71%. However, the general strong improvement in shoulder function and pain relief is consistent with the long-term results of El-Azab et al. [48•], who reported a satisfaction rate of 86%. In this cohort of 115 shoulders, the failure rate of the open LDT was 10%, and 4% needed reoperation to implant a RTSA [48•]. Although substantial progression of OA was observed in 11% of the patients 10 years postoperatively [20••], the necessity of RTSA can be reportedly delayed [54•]. Older patients and patients with a transfer after a failed RC repair had a slight but significant inferior clinical outcome compared with the patients with a primary LDT. Those shoulders that had the LDT in combination with subscapularis repair showed actually no difference in the clinical outcome compared with those with an intact subscapularis.

Although no long-term results of the arthroscopic-assisted LDT are available, short-term results are comparable with the open techniques with significantly improved shoulder function and reliable pain relief [21•]. Complications of the arthroscopic and open techniques are comparably low and include postoperative hematoma, frozen shoulder, nerve injuries, infection, and failure of the tendon transfer [21, 55]. In one study, the overall complication rate of 258 pooled arthroscopic-assisted LDTs was 7.3%, including tendon rupture (2.7%), deep infection (2.3%), postoperative hematoma (2%), and transient brachial plexus palsy (0.4%) [21•].

## Conclusions

The LDT (open or arthroscopically assisted) is a reliable treatment option with substantial and durable improvement of pain and shoulder function for patients with massive, irreparable posterosuperior RCT with disabling loss of active external rotation, with or without diminished overhead function, and without glenohumeral osteoarthritis. The long-term failure rate is about 10%, and 4% of patients need revision to RTSA after 10 years. Careful patient selection is of tremendous importance in the success of the LDT. Teres minor degeneration, associated irreparable subscapularis tears, a high CSA, and advanced age are risk factors for unsatisfactory outcome.
